# Elongated Riboflavin‐Producing *Shewanella* *o* *neidensis* in a Hybrid Biofilm Boosts Extracellular Electron Transfer

**DOI:** 10.1002/advs.202206622

**Published:** 2023-01-29

**Authors:** Juntao Zhao, Feng Li, Shutian Kong, Tao Chen, Hao Song, Zhiwen Wang

**Affiliations:** ^1^ Frontier Science Center for Synthetic Biology Tianjin University Tianjin 300072 P. R. China; ^2^ Key Laboratory of Systems Bioengineering (Ministry of Education) Tianjin University Tianjin 300072 P. R. China; ^3^ School of Chemical Engineering and Technology Tianjin University Tianjin 300072 P. R. China

**Keywords:** biofilms, cellular morphology, extracellular electron transfer, flavin

## Abstract

*Shewanella oneidensis* is able to carry out extracellular electron transfer (EET), although its EET efficiency is largely limited by low flavin concentrations, poor biofilm forming‐ability, and weak biofilm conductivity. After identifying an important role for riboflavin (RF) in EET via in vitro experiments, the synthesis of RF is directed to 837.74 ± 11.42 µm in *S. oneidensis*. Molecular dynamics simulation reveals RF as a cofactor that binds strongly to the outer membrane cytochrome MtrC, which is correspondingly further overexpressed to enhance EET. Then the cell division inhibitor *sulA*, which dramatically enhanced the thickness and biomass of biofilm increased by 155% and 77%, respectively, is overexpressed. To reduce reaction overpotential due to biofilm thickness, a spider‐web‐like hybrid biofilm comprising RF, multiwalled carbon nanotubes (MWCNTs), and graphene oxide (GO) with adsorption‐optimized elongated *S. oneidensis*, achieve a 77.83‐fold increase in power (3736 mW m^−2^) relative to MR‐1 and dramatically reduce the charge‐transfer resistance and boosted biofilm electroactivity. This work provides an elegant paradigm to boost EET based on a synthetic biology strategy and materials science strategy, opens up further opportunities for other electrogenic bacteria.

## Graphical abstract

A spider‐web‐like hybrid biofilm comprising riboflavin (RF), multiwalled carbon nanotubes (MWCNTs), graphene oxide (GO) with adsorption elongated *S. oneidensis*, achieves a 77.83‐fold increase in power (3736 mW m^−2^).

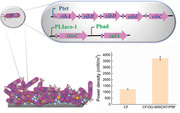

## 1 Introduction

Bi‐directional extracellular electron transfer (EET) between electroactive microorganisms and their surroundings are attracting intense attention owing to their potential abilities for achieving sustainable value‐added resources, i.e., bioelectricity, biofuels, and chemicals, from organic and inorganic waste in bioelectrochemical systems (BESs). These include microbial fuel cells (MFCs),^[^ ^1^ ^]^ microbial electrolysis cells,^[^ ^2^ ^]^ and microbial electrosynthesis systems.^[^ ^3^ ^]^ This provides a potential solution for two major problems facing the world today, environmental pollution and energy shortage. The EET process is a key factor in determining the performance of BESs.

*S. oneidensis* MR‐1 shows great promise as a well‐studied EET strain in bio‐remediation of contaminated wastewater and environmental energy recovery because of its robust growth, remarkable anaerobic versatility, and widespread distribution.^[^ ^4^ ^]^ Generally, *S. oneidensis* cells are attached to the electrode in a biofilm matrix to achieve EET through a series of direct or indirect electron transfer processes (Figure S1, Supporting Information). Overall, EET is divided into the following steps i) electrons are transported from the cytoplasmic membrane (CymA), through the periplasm (STC and FccA) and across the outer membrane (OmcA‐MtrABC);^[^ ^5^ ^]^ ii) electron shuttles (e.g., flavins) can then diffuse into the medium and are thus able to pass on the electrons to an external electrode^[^ ^6^ ^]^ and iii) the microbe‐electrode interface reaction occurs through biofilm formation.^[^ ^7^ ^]^

Research efforts have largely focused on enhancing EET by accelerating the synthesis and transmission of electron shuttles (e.g., flavins)^[^ ^8^ ^]^ and regulating biofilm formation.^[^ ^9^ ^]^ For example, genetic engineering of MR‐1 has resulted in flavin concentrations of 1.33–39.7 µm.^[^ ^8^, ^10^ ^]^ Modification of extracellular structural components (eDNA, extracellular polysaccharides and proteins) and intracellular regulatory factors (quorum sensing components and *c*‐di‐GMP) has been used to improve biofilm formation.^[^ ^11^ ^]^ Despite these strategies, the power density of the MFCs to date has hit a plateau ranging from several hundreds to thousands of milliwatts per square meter, level that are still lower than conventional chemical fuel cells by one to two orders of magnitude. The EET efficiency from *Shewanella* MFCs has been too low for practical applications^[^ ^12^ ^]^ largely because of low levels of flavins synthesis^[^ ^13^ ^]^ and relatively poor biofilm formation^[^ ^14^ ^]^ and conductivity.^[^ ^15^ ^]^

Recently, different types of anode materials have been developed to achieve high surface, good electron transfer, and bacterial adhesion for improving the efficiency of energy generation, which has become a new research interest in the field of MFCs.^[^ ^16^ ^]^ Various carbon‐based nanoparticles, such as graphene and carbon nanotubes (CNTs), have shown great potential for anode additives in MFCs. Composite materials based on graphene/CNTs have been prepared by different means, including chemical vapor deposition,^[^ ^17^ ^]^ electrophoretic deposition,^[^ ^18^ ^]^ microwave energy‐assisted unzipping process,^[^ ^19^ ^]^ coating membrane,^[^ ^20^ ^]^ and in situ chemical reduction.^[^ ^21^ ^]^ For example, nitrogen doped CNTs can achieve a power density of 1137 mW m^−2^ by doping reduced graphene oxide and polyaniline as anodes of fuel cells.^[^ ^22^ ^]^ A nitrogen‐doped MWCNT@GONR (N‐MWCNT@GONR) were synthesized through a microwave energy‐assisted unzipping process with a core–shell structure significantly reduced the charge transfer resistance, resulting in MFC output power of up to 3444 mW m^−2^.^[^ ^19^ ^]^ These studies have made significant progress in developing functional electrodes. However, the process of making functional electrodes (e.g., electrophoretic deposition) are relatively complex, usually time‐consuming, labor intensive and require specific instrument.

In this paper, we first verified that riboflavin (RF) as free electron shuttles play a decisive role in EET via in vitro experiments, and then molecular dynamics simulation further revealed RF as cofactor that binds stronger to outer membrane *c‐*type cell cytochromes (*c*‐Cyts) protein MtrC. We then enhanced EET mediated free‐RF and bound‐RF by directional synthesis of RF based different combinations of structural genes (RF operons) and regulatory elements (e.g., promoters and plasmids) and overexpression of MtrC. We further enhanced the biofilm formation ability by overexpressing cell division inhibitor *sulA*. Finally, to further boost biofilm electroactivity, a spider‐web‐like hybrid biofilm comprising RF, multiwalled carbon nanotubes (MWCNTs), graphene oxide (GO) with adsorption elongated *S. oneidensis*, achieved a 77.83‐fold increase in power (3736 mW m^−2^,) relative to MR‐1 and dramatically reduced the charge‐transfer resistance. This study demonstrates a facile and effective strategy to boost EET of *S. oneidensis* MR‐1.

## 2 Results

### 2.1 Impact of Different Flavins on EET of *S. oneidensis* In Vitro

**Figure 1:**
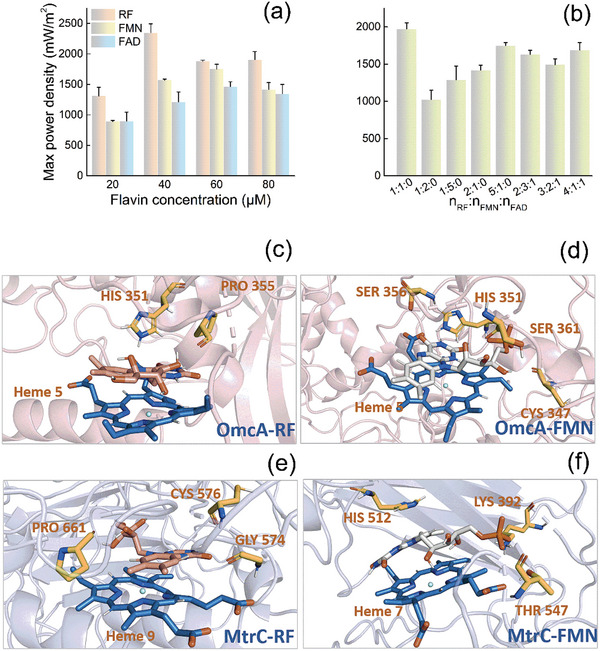
Power density of MFCs with different kinds and different concentrations of flavins. a) Power density of MFCs in the presence of a single flavin at the indicated concentration Flavin concentration. b) Power density of MFCs with different molar ratios of the three flavins. c–f) Molecular dynamics simulations between outer‐membrane *c* type cytochromes proteins (*c*‐Cyts) OmcA c,d) and MtrC e,f) and RF c,e) and FMN d,f). The error bars represent the standard deviation from three independent experiments.

“Flavins” is a general term for a class of yellow organic compounds derived from isoalloxazine rings based on pteridine. The main members are RF, flavin adenine dinucleotide (FAD) and flavin mononucleotide (FMN). RF is a precursor of FMN and FAD biosynthesis. In *S. oneidensis*, flavins, mainly free RF and FMN, are electron shuttles that mediate a two‐electron transfer reaction.^[^ ^23^ ^]^ Additionally, RF and FMN associate with MtrC and OmcA as cofactors to mediate two single‐electron transfer reactions (Figure S1, Supporting Information).^[^ ^24^ ^]^ In contrast, FAD is secreted into the periplasmic space through the natural inner membrane FAD transporter *bfe* and then is hydrolyzed into FMN by *ushA*.^[^ ^25^ ^]^ Whether FAD can play the same role as RF and FMN in the extracellular environment has not been reported. It is also unknown which flavin is most advantageous for EET and whether there is a synergistic effect among the three flavins during EET. To explore the role of free flavins during the process of EET, we conducted experiments using flavins as electron shuttles in MFCs (Tables S3 and S4, Supporting Information). With the addition of RF to a final concentration of 40 µm, the power density reached its maximum (2350 ± 146 mW m^−2^, *n* = 3), which was at least 1.2–2.6‐fold higher as compared to with the concentration of other flavins, respectively (**Figure** **1**a). In contrast, a concentration of 60 µM for each of the other two flavins (FMN and FAD) of resulted in a maximum power value 1753 ± 76 and 1463 ± 81 mW m^−2^, respectively (*n* = 3) (Figure 1a). These results indicated that RF is more effective than FMN and FAD at the same concentration during EET process and RF is a key factor in EET.

To further analyze whether there is synergy among the three flavins during EET, we added the three flavins at different molar ratios but with a total flavin concentration maintained of 40 µm. The power density was at a maximum (1972 ± 82 mW m^−2^, *n* = 3) with a molar ratio of 1:1 RF/FMN in the absence of FAD (Figure 1b). After adding FAD, when the molar ratio of RF, FMN, and FAD was 4:1:1, the power density reached 1688 ± 100 mW m^−2^ (*n* = 3) (Figure 1b). Thus, RF and FMN play a more important role than FAD in this system. Meanwhile, we can see that the maximum power value of adding only RF to the MFC was higher than that of adding two or three flavins. Overall, the above results show that RF, among these three flavins, plays a decisive role in the EET process, followed by FMN.

Flavins (RF and FMN) are reduced while serving as cofactors by the Mtr respiratory pathway in MR‐1 during EET.^[^ ^24^ ^]^ To further investigate the importance of RF and FMN as cofactors during EET, we carried out molecular dynamics simulations to explore the interfacial relationships between outer‐membrane cytochromes (OmcA and MtrC) and flavins (RF and FMN), respectively (Figure 1c–f). Structure display of proteins and small molecules was shown in Figure S2 (Supporting Information). First, we screened for an optimal configuration using molecular docking (Figure S3, Supporting Information) and calculated the binding affinity. OmcA interacted with both RF and FMN through hemes 5 and 7, respectively (Figure 1c,d), with the binding affinity of the RF‐OmcA complex slightly higher than that of FMN‐OmcA along with a shorter flavin‐heme distance (Table S5, Supporting Information). In addition, two identical docking positions were identified for RF and FMN on MtrC close to hemes 9 and 7, respectively (Figure 1e,f), with higher affinity for RF‐MtrC complex than that of the FMN‐MtrC complex (Table S5, Supporting Information). Both parameters indicated a stronger interaction for OmcA and MtrC with RF than FMN. The best configurations, i.e., MtrC‐RF heme 9, MtrC‐FMN heme 7, OmcA‐RF heme 5, and OmcA‐FMN heme 5, were selected for molecular dynamics simulation using Gromacs software.^[^ ^26^ ^]^ RF had a strong affinity for MtrC with a binding free energy of −150.667 kJ mol^−1^, which was lower than that associated with the other three configurations (Table S6, Supporting Information), indicating that MtrC was more closely bound to RF. In a world, RF is more advantageous than FMN with respect to the EET process and provide guidance for our subsequent engineering of strains.

### 2.2 Enhancing EET Mediated by Free‐RF and Bound‐RF by Directed Synthesis of RF and Overexpression of *c*‐Cyts

**Figure 2:**
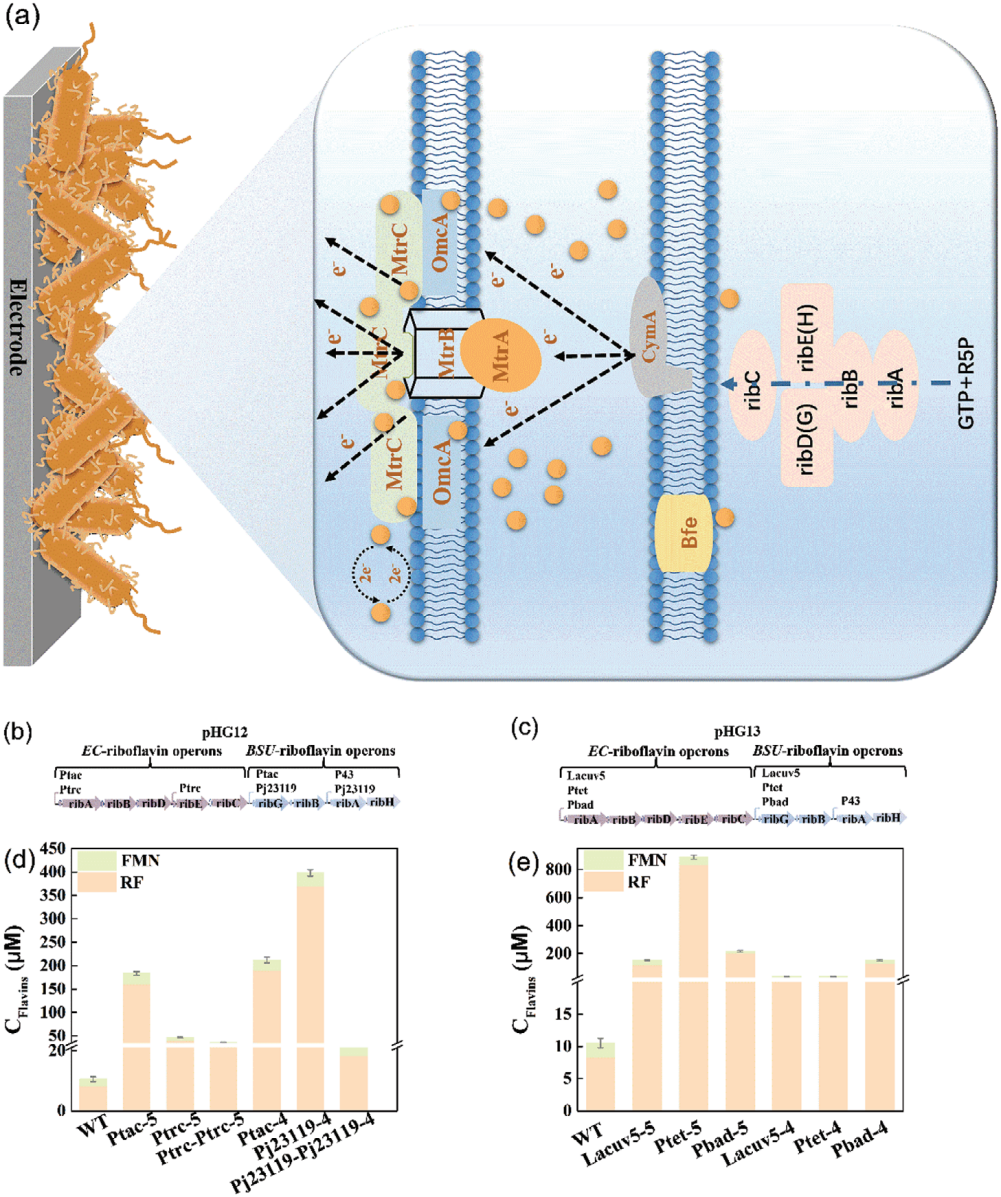
Enhanced RF production by directed synthesis of RF based on multiple strategies. a) A schematic diagram of the components involved in promoting RF synthesis, coupled with overexpression of cytochrome MtrC, which led to greater electricity generation. b,c) Schematic diagram of promoters of different strengths for overexpression of RF operon from two different sources (*ribGBAH, BSU*; *ribABDEC, EC*) in plasmids pHG12 b) and pHG13 c). d,e) Histogram of flavins production after transfection with the pHG12‐based plasmids shown in (b) d) or pHG13‐based plasmids shown in (c) e). Strains were based on Ptac‐5 or Ptac‐4. Independent data from MR‐1 are shown for comparison. “Ptrc‐5” refers to the replacement of the Ptac promoter to Ptrc on the basis of strain Ptac‐5. “Ptrc‐Ptrc‐5” refers to addition the Ptrc promoter in front of *ribE* in on the basis of strain Ptrc‐5. “Pj23119‐4” refers to the replacement of the Ptac promoter to Pj23119 on the basis of strain Ptac‐4 “Pj23119‐Pj23119‐4” refers to the replacement of the P43 promoter in front of *ribG* to Pj23119 in on the basis of strain Pj23119‐4. The error bars represent the standard deviation from three independent experiments.

AS WT MR‐1 is able to secrete RF at a concentration of ≈8 µm, there is ample room for improvement in the level of RF. We thus directed the synthesis of RF through different combinations of structural genes (RF operons, **Figure** **2**a) and regulatory elements (e.g., promoters and plasmids; Figure 2b,c). Different RF operons (*BSU*‐*ribGBAH* and *EC*‐*ribABDEC*, see Table S7 (Supporting Information) for details) were individually overexpressed with the low‐copy‐number plasmid pHG12 under the inducible Ptac promoter (1 mm IPTG) (Figure 2b), producing 211.76 ± 6.36 µm (*n* = 3) and 183.38 ± 3.66 µm (*n* = 3) flavins in Ptac‐4 and Ptac‐5, respectively, which was 17–20‐fold higher than that of WT MR‐1 (≈10.50 ± 0.74 µm, *n* = 3), respectively (Figure 2d). Subsequently, to promote the coordinated expression of genes, promoter engineering was carried out to improve the RF levels, resulting in a higher flavin concentration (398.10 ± 6.83 µm, *n* = 3) when the higher strength promoter Pj23119^[^ ^27^ ^]^ was used in a strain based on Ptac‐4 (Figure 2d). To further increase the expression of the gene for enhancing the concentration of RF, the higher‐copy‐number plasmids pHG13 was used to overexpress the different RF operons under inducible promoters of different strengths—low, Lacuv5; medium, Ptet, and high, Pbad (Figure 2c).^[^ ^27^ ^]^ Ptet‐5 resulted in flavin generation of 888.99 µm (RF 837.74 ± 11.42 µm, FMN 51.25 ± 2.72 µm, *n* = 3), which was 82‐fold higher than that of MR‐1 in LB medium (Figure 2e). To the best of our knowledge, this flavin concentration is the highest among reported engineered *S. oneidensis* strains (see Table S8, Supporting Information). Taken together, these results suggest that RF production can be effectively increased through operons engineering, promoter engineering, and plasmid engineering.

**Figure 3:**
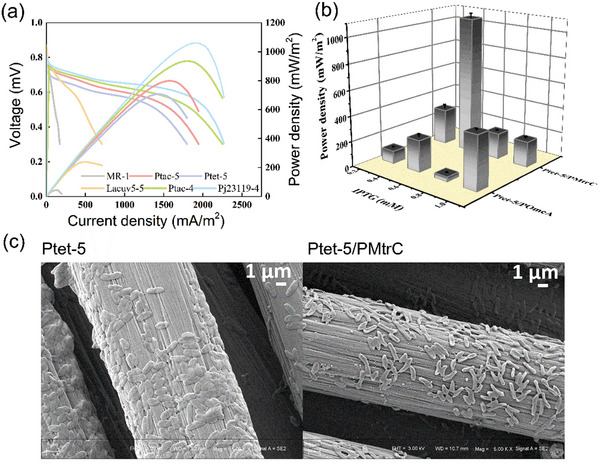
Bioelectrochemical characterization of engineered *Shewanella* strains with different levels of RF production. a) MFC polarization curves and power density output curve. b) Effect of IPTG‐induced expression of *c* type cytochrome proteins on the power performance of MFCs. c) SEM of biofilm morphology at electrode surface of strains Ptet‐5 in the absence (Ptet‐5) and presence (Ptet‐5/MtrC) of *mtrC* overexpression. The error bars represent the standard deviation from three independent experiments.

To investigate the effect of different levels of RF on EET, we next carried out an electrochemical analysis of these engineered strains. As compared with the MFCs inoculated with MR‐1, the MFCs inoculated with the engineered strains all showed higher potential power production performance. Among them, strains Pj23119‐4 (370.91 ± 5.58 µm RF) and Ptet‐5 (837.74 ± 11.42 µm RF) showed higher output voltages of 530 ± 2 mV (*n* = 3) and 415 ± 1 mV (*n* = 3), respectively (Figure S4a, Supporting Information), and a steeper slope, which corresponds to the internal resistance in MFCs than MR‐1 (**Figure** **3**a). Strikingly, the larger reduction in the current response for strains Pj23119‐4 and Ptet‐5 than MR‐1 noted in the cyclic voltammetry (CV) analysis indicated their faster EET rate (Figure S4b, Supporting Information). The maximum power density of strains Ptet‐5 and Pj23119‐4 was 707 ± 18 mW m^−2^ (*n* = 3) and 1061 ± 27 mW m^−2^ (*n* = 3), which were ≈15 times and ≈22 times higher than that of the MR‐1 strain (48 ± 6 mW m^−2^, *n* = 3), respectively (Figure 3a).

One odd phenomenon is that strain Ptet‐5, which had a high RF concentration, did not exhibit a higher power density relative to the other modified strains. We suspect that this was due to the high levels of RF being underutilized, as studies have shown that *c*‐Cyts (MtrC and OmcA) either can be reduced or can use flavins (RF and FMN) as cofactors.^[^ ^28^ ^]^ Thus, we overexpressed *omcA* and *mtrC* in separate engineering strains derived from Ptet‐5. As *c*‐Cyts are membrane proteins, high levels of overexpression would be toxic to the host (Figure S4c, Supporting Information). We thus used a range of Isopropyl‐beta‐D‐thiogalactopyranoside (IPTG) concentrations (0.25–1 mm) to control the expression level of *c*‐Cyts. The MtrC‐expressing strain Ptet‐5/PMtrC produced a maximum power density of 1073 ± 35 mW m^−2^ (*n* = 3), 1.48 times that of the control strain, in the presence of 0.5 mm IPTG (Figure 3b). We next measured the CV curves and polarization curves of MFCs inoculated with the engineered strains (Figure S5, Supporting Information). The results further verified that the high concentration of RF in the engineered strains bound to MtrC, in addition to OmcA, as a cofactor to promote EET.

Although the EET efficiency was significantly improved by directed synthesis of RF and overexpression of *c*‐Cyts, the output power was still lower than expected. Scanning electron microscopy (SEM) imaging analysis showed that each carbon rod was covered by only a handful of cells at the anode of MFCs inculcated with strain Ptet‐5/PMtrC (Figure 3c). Such thin electroactive biofilms, with their small biomass, are associated with low efficiency of extracellular electron transport.^[^ ^29^ ^]^ Thus, to further improve the power generation performance, it was necessary to improve the ability of these engineered strains to form a biofilm.

### 2.3 Improving Biofilm Formation via the Regulation of Cell Morphology in Engineered *S. oneidensis* Cells

**Figure 4:**
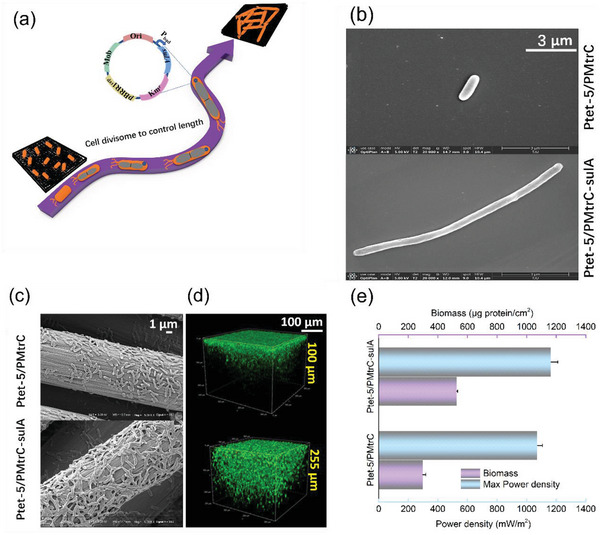
The effect of cell morphology on biofilm formation. a) Schematic diagram of cells with different growth patterns attached to an electrode. *sulA*, an SOS‐inducible protein that prevents FtsZ from polymerizing to form the Z‐loop, was selected for overexpressing under the control of the arabinose‐inducible promoter Pbad to change in cell morphology. b) SEM of the parental Ptet‐5/PMtrC and the further engineered strain that overexpression of *sulA* (Ptet‐5/PMtrC‐sulA). Cells were grown in LB medium at 30 °C and 200 rpm for 12 h under aerobic conditions. c) SEM of the biofilm at the electrode surface that results form Ptet‐5 and Ptet‐5/PMtrC‐sulA. d) Confocal laser scanning microscopy of cells in the absence (upper) and presence (lower) of *sulA* overexpression on the electrode surface. e) The biomass (upper) and maximum power density (lower) of strains Ptet‐5 and Ptet‐5/PMtrC‐sulA when used to generate a biofilm on the electrode surface. The error bars represent the standard deviation from three independent experiments.

As the ability to form biofilms is related to the resulting electrocatalytic activity, we hypothesized that a change in cell morphology through morphological engineering might improve the adsorption of cells on the electrode and thus improve electrocatalytic activity. The cell morphology is maintained by a variety of genes related to cell division, cell wall synthesis, and cytoskeleton formation, such as *ftsZ* encoding tubulin related GTPase and *mreB* encoding actin like protein.^[^ ^30^ ^]^ It has been proved that the interaction between *sulA* and *ftsZ* inhibits the formation of FtsZ ring, leading to the rod like extension into filament, which increases the cell size and intracellular space.^[^ ^31^ ^]^ A schematic diagram of our approach is shown in **Figure** **4**a. To test the validity of our conjecture, we overexpressed different genes related to cell morphology (e.g., *sulA*, *mreB*, and *ftsZ*). Compared with the control strain, the engineered strains that overexpressed the genes related to cell morphology grew slower, while expression of *sulA* resulted in the greatest effect on biofilm formation and EET enhancement (Figure S6, Supporting Information). Therefore, *sulA* was further overexpressed in engineered strain Ptet‐5/PMtrC under the control of the arabinose‐inducible promoter Pbad. SEM images showed that the cells of the parental strain were 1–3 µm in length, whereas the further engineered strain with *sulA* overexpression showed a cell elongation effect, with fivefold increases in cell length, thus resulting in a “spaghetti‐like” growth after 12 h of incubation (Figure 4b).

We then analyzed the resulting electroactive biofilms that formed on the surface of the anodes. SEM indicated that there was a large number of slender cells attached to the anode in a certain arrangement (Figure 4c). Such biofilms were measured to have 527 ± 7 µg protein cm^−2^ (*n* = 3) in strain Ptet‐5/PMtrC‐sulA, an 89% increase relative to the control cells (298 ± 21 µg protein cm^−2^, *n* = 3) (Figure 4e). Similarly, the confocal laser scanning microscopy image showed a denser biofilm with a much higher number of bacteria at the anode with Ptet‐5/PMtrC‐sulA (Figure 4d). The elongated cells showed very distinct growth and cell arrangement patterns on the electrode surfaces relative to the control cells (Figure 4c). Most of the elongated cells showed a spiraling and twisted arrangement on the electrode surfaces. Bioelectrochemical analysis indicated that the MFCs inoculated with the engineered strains of Ptet‐5/PMtrC‐sulA achieved a higher power density upon induction by 3 mm arabinose (1165 ± 46 mW m^−2^, *n* = 3), a 1.03‐fold increase relative to that of control strain Ptet‐5/PMtrC (Figure 4e).

Although the elongated cells formed a denser biofilm, the increase in power was not as high as expected. An impedance spectrum analysis showed that strain Ptet‐5/PMtrC‐sulA had a charge‐transfer resistance values (*R*_ct_) of 1600 ohms (Figure S7, Supporting Information). This may be because the thicker biofilm increased the reaction overpotential, hindered mass diffusion of the substrate, and/or resulted in poor biocompatibility, all of which may have affected power generation.

### 2.4 Improving Biofilm Conductivity by Forming Spider‐Web‐Like Artificial Hybrid Biofilm

**Figure 5:**
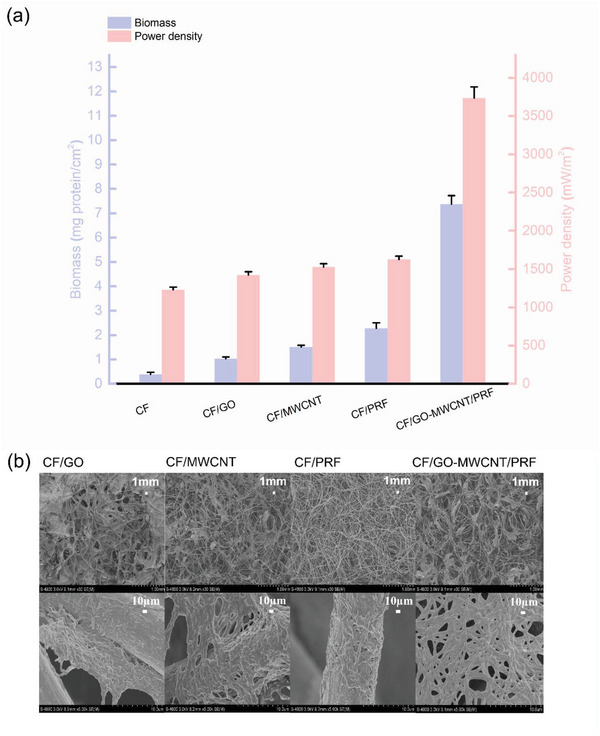
The effect of the modified electrodes on biofilm formation. a) Procedure for the composite modification electrode. The strain Ptet‐5/PMtrC‐sulA that was used for inoculation. b) SEM images of the modified electrodes and the resulting biofilms on the CF/GO, CF/MWCNT, CF/PRF, and CF/GO‐MWCNT/PRF electrodes. The upper images show the structure itself, whereas the lower images show the resulting biofilm on that structure. The error bars represent the standard deviation from three independent experiments.

To reduce any reaction overpotential and improve MFC performance, we developed a GO‐based electrode. The honeycomb‐like structure of GO can increase the specific surface area for biofilm formation, promote the interaction between microorganisms and substrates, increase active sites and reduce reaction overpotential.^[^ ^32^ ^]^ However, the pristine surface of carbon felt is relatively inert, making it difficult to support GO particles uniformly, which often leads to agglomeration of GO nanoparticles.^[^ ^33^ ^]^ MWCNTs readily form porous structures that can act as a support for developing better structures as well as high entanglement and electrical conductivity for modified GO electrodes. These attributes in turn facilitate faster substrate transport, which provides enhanced electron transfer. One disadvantage of nanomaterial‐modified electrodes is their poor biocompatibility. In contrast, flavins possess not only a high electron transfer capacity but also good biocompatibility. The RF was further adsorbed on modified GO‐MWCNT electrodes by electro‐aggregation to form aggregated PRF with the effective biocompatibility of polyriboflavin interface (Figure S8 (Supporting Information); and **Figure** **5**a). CV curves showed that there are two pairs of redox peaks at −0.363/−0.497 and −0.22/−0.269 V, respectively (Figure S8, Supporting Information). A sharp oxidation peak appeared at the potential of +1.497 V, indicating that the isoalloxazine ring of riboflavin was formed due to the free radicals of nitrogen or carbon atoms. All oxidation peaks increased with the increase of cycle times, indicating that poly‐riboflavin gradually increased on the surface of CF electrode.

After modification, the appearances of the functional anodes had changed dramatically. SEM images showed that the modified electrodes exhibited greater surface roughness. For example, GO resulted in a sheet‐like structure, whereas MWCNTs were intertwined with one another to form a network‐like structure (Figure 5b).

To evaluate the effectiveness of our approach, we further tested the compactness the biofilms. The SEM images indicated that i) a layer of strip‐like cells spread over the GO sheets, which may have acted as nets to capture *S. oneidensis* like a “fishing process” and enhance *π*–*π* interactions between the GO sheets and bacteria to increase the conductivity of the CF/GO electrode. ii) The CF/MWCNT electrode, due to the “armrest” support of MWCNT, the cells are like “filaments” holding hands and clinging to the MWCNT, such as the creeper can cling to the wall surface, forming a tight and strong mesh biofilm. iii) The elongated cells were tightly wrapped on the CF/PRF surface. iv) The composite electrode CF/GO‐MWCNT/PRF resulted in the densest and tightly adherent biofilm, some like “spider web” distribution, layer upon layer, some like “roots” coiled, tangled (Figure 5b).

In addition, we found that GO was indeed reduced to rGO according to a spectroscopic technology analysis based on several lines of evidence. First, the carbon 1s (C1s) spectra from X‐ray photoelectron spectroscopy showed an obvious oxidation (CO) peak (≈286.7 eV) and a stronger CC peak (≈284.6 eV), indicating that GO was reduced to rGO (Figure S9, Supporting Information).^[^ ^34^ ^]^ Second, O—H stretching in GO had an obvious redshift from ≈3400 to ≈3300 cm^−1^, which may be attributed to *π*–*π* interactions between rGO and flavin.^[^ ^35^ ^]^ Third, the peak at ≈1700 cm^−1^ (due to the stretching vibration of CO) and ≈1380 cm^−1^ (due to the bending vibration of OH) decreased after GO reduction (Figure S10, Supporting Information).^[^ ^36^ ^]^

**Figure 6:**
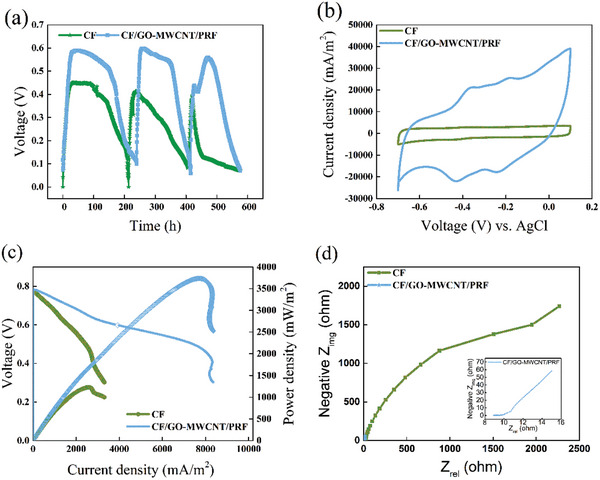
Performance comparison of *Shewanella* MFCs with different anodes. The strain Ptet‐5/PMtrC‐sulA that was used for inoculation in MFCs. a) Voltage output of MFCs with different anodic materials. b) CV curves of MFCs with different anodic materials. c) *I*–*V* curves (left axis) and power polarization curves (right axis) for MFCs with two different anode materials. d) Nyquist plots of electrochemical impedance spectroscopy scanned at open circuit potential of 0.01–100 000 Hz with a 10 mV perturbation. The error bars represent the standard deviation from three independent experiments.

Bioelectrochemical analysis was used to validate the MFC performance of the modified electrodes. The output voltages of all MFCs with different anodic materials have repeatable voltage outputs that can last about 600 h after 3 cycles of operation, indicating successful establishment of a functional MFC (**Figure** **6**a; and Figure S11a, Supporting Information). Strikingly, the CF/GO‐MWCNT/PRF electrode showed a higher catalytic current and a maximum power density of 3736 ± 147 mW m^−2^ (*n* = 3), which is higher 1.30 to 2.04‐fold higher that of other anodes (Figure 6b,c; and Figure S11b,c, Supporting Information). The higher power generation of the CF/GO‐MWCNT/PRF may be due to the increased number of bacteria and the decrease in the internal resistance. The total protein level of the biofilm attached to the CF/GO‐MWCNT/PRF electrode was 7.78 ± 0.35 mg cm^−2^, which was 3.83–15.88 times that of the other electrodes (Figure 5a). Similarly, the *V*–*j* curve and the Nyquist plots showed that the CF/GO‐MWCNT/PRF electrode had a lower internal ohmic resistance and interfacial charge‐transfer resistance (*R*_ct_ = 15.7 ohms), respectively (Figure 6c,d; and Figure S11c,d, Supporting Information). Thus, the CF/GO‐MWCNT/PRF electrode led to a decrease in the ohmic resistance against anodic interfacial electron transfer.

## 3 Discussions

Although previous studies have revealed the importance of flavins in EET,^[^ ^37^ ^]^ it was not clear how important the three most common flavin types are with respect to the final power output of a MFC and whether there is synergy effect among the different flavins. RF and FMN are equally effective as electron shuttles when added at concentration of 4–5.5 µm of flavins (RF or FMN) to MFCs.^[^ ^38^ ^]^ In contrast, the present study revealed for the first time that RF plays a more decisive role in EET than FMN and FAD based on rationally designed MFCs with adding flavins.

In practice, adding flavins to MFCs is not advisable because of the added cost. Researchers raised the flavins concentration via genetic engineering of MR‐1 to 1.33–39.7 µm flavins (mainly FMN).^[^ ^8^, ^10^ ^]^ However, the RF levels in these strains remain in the lower range. Herein we directed synthesis of RF using RF operons from different sources, promoters of different strengths, and plasmids with different copy numbers, which led to the biosynthesis of 888.99 µm flavins, 94% of which consisted of RF (Figure 2e). This is 21.4‐ and 667‐fold higher than values reported using *S. oneidensis* MR‐1 cells in LB^[^ ^8^ ^]^ and in mineral medium,^[^ ^10^ ^]^ respectively. To the best of our knowledge, this is the highest titer of RF in engineered *S. oneidensis*. Strangely, the power density was reduced by increasing RF production to 837.74 ± 11.42 µm. We guess that reasons are as follows: i) compared with the experiment of adding RF in vitro, the substrate could be mainly oxidized to produce electrons. While in the high RF‐producing strain, the substrate was partly used to produce RF and partly used to produce electrons. ii) the transport of RF is dependent on ATP.^[^ ^39^ ^]^ Energy‐coupling factor are a recently discovered family of primary active transporters for RF. ATP binding dissociates the complex between the ECF module and the riboflavin‐binding protein, RibU, thus allowing the released S subunit, a membrane‐embedded substrate‐binding subunit, to capture RF.^[^ ^40^ ^]^ In addition, conversion of RF into FMN and FAD is catalyzed a single bifunctional protein, FAD synthetase transforms RF and ATP into FMN, and FMN into FAD using a second molecule of ATP.^[^ ^41^ ^]^ iii) studies have shown the role of RF as electron shuttle varied with dissolved oxygen values^[^ ^42^ ^]^ and each flavin has a unique pH dependency to compensate for the EET enhancement.^[^ ^43^ ^]^

We also noted that EET was further enhanced when MtrC was overexpressed in RF‐producing *S. oneidensis* (Figure 3b). This suggests a stronger interaction of RF as cofactor with MtrC than other configurations, which is consistent with our molecular dynamics simulations results. In conclusion, we demonstrate that the synthesis of RF and overexpression of the corresponding *c*‐Cyts are an effective strategy for improving EET mediated by free‐RF and bound‐RF.

As previous attempts to promote EET by biofilm formation based on the modification of extracellular structural components and intracellular regulatory factors^[^ ^44^ ^]^ had limited success, we followed a novel strategy that focused on cell morphology. We hypothesized that changes effected through morphological engineering could improve the adsorption of cells on the electrode. *sulA*, an SOS‐inducible protein that prevents FtsZ from polymerizing to form the Z‐loop,^[^ ^45^ ^]^ was selected for overexpressing in engineered strains Ptet‐5/PMtrC. The morphology of the resulting cells showed a marked elongation as compared with control cells and formed a thicker and denser biofilm. Electricity production via these cells was 1165 ± 46 mW m^−2^,which is 7 times than overexpressed *ydeH*, a *c*‐di‐GMP biosynthesis gene in *S. oneidensis* (167.6 mW m^−2^);^[^ ^44c^ ^]^ 21 times than expressed curli hybrid proteins on the *S. oneidensis* cell surface (55 mW m^−2^).^[^ ^46^ ^]^ In conclusion, for the first time, a morphologic engineering strategy was used to promote biofilm formation, clarifying that cell enlargement is beneficial to the formation of biofilms and thus improve EET in this study. In the future, cell size can be further precisely regulated to study its effect on biofilm formation and electricity production.

Both GO and CNTs, with large surface area, and good electrical conductivity, they have been widely used to modify anode surfaces to improve anode performance.^[^ ^47^ ^]^ However, CNTs are poorly soluble in water and usually require surfactants to disperse, which impairs the excellent electrochemical performance. The strong van der Waals forces between GO sheets promote their agglomeration during the reduction process, which in turn affects GO effect. The oxygen‐containing functional groups on the edge of GO sheets can be involved in the synthesis various soft materials through chemical reactions, so that GO can be used as a surfactant to achieve CNT dispersion through *π*–*π* stacking.^[^ ^48^ ^]^ Therefore, we used carbon felt as the anode base in combination with modified GO with a large surface area, MWCNTs with good conductivity and high concentrations of RF adsorbed by electro‐aggregation to improve biocompatibility. The resulting modified electrode greatly promoted power generation (3736 mW m^⁠−2^), resulting in one of the highest power densities reported so far in *Shewanella* (Table S9, Supporting Information). Our system has two advantages. i) The electrode comprises three different materials to give full play to their respective advantages and complement each other's strengths and weaknesses. ii) The electrodes are easy to generate and simple to operate. Thus, these modified electrodes should lead to great improvements of electricity production and should reduce charge transfer resistance.

The above‐mentioned, interface engineering strategies, including the use of conductive nanomaterials to improve the interface properties of electroactive microorganisms (EAMs) and electrodes, and the use of synthetic biology techniques to modify EAMs, can effectively boost the EET efficiency. We found that the improvement of MFC output performance by constructing 3D electrodes modified by conductive nanomaterials could be much greater than that by engineered EAMs. Indeed, the electrode modified by RF, MWCNTs, and GO dramatically reduced the charge‐transfer resistance and boosted biofilm electroactivity. This is mainly due to a spider‐web‐like hybrid biofilm formation comprising RF, MWCNTs, and GO with adsorption‐optimized elongated *S. oneidensis*. For microorganisms, as the electroactive catalyst in MFC, in addition to specific biochemical or metabolic functions, their EET mechanism and efficiency, as well as the ability to form electroactive biofilms, are essential for their interaction with electrodes.^[^ ^49^ ^]^ Therefore, constructing electrocatalytic cell factories with powerful EET capabilities is of uppermost priority. For electrodes, several inherent characteristics are critical to the adhesion of microorganisms, as well as simultaneous electron transfer,^[^ ^50^ ^]^ such as conductivity and biocompatibility.^[^ ^50^ ^]^ In a word, constructing 3D hybrid biofilm will have a decisive impact on the interface interaction between nanomaterials and microorganisms, thus affecting the EET efficiency.

In conclusion, our results demonstrate the key role of RF in EET in *Shewanella*, which provides an important foundation reference point for further research on the EET mechanism and for rational engineering of *Shewanella*. These findings offer insights into the process of cellular morphologyenhanced EET via regulating biofilm formation, which holds promise for future power generation systems. This new combinatorial engineering strategy to improve EET based on a synthetic biology approach and materials science innovations provides a foundation and guidance for subsequent research on other electrogenic bacteria.

## 4 Experimental Section

For more detailed information see the Supporting Information.

### Plasmid Construction and Electroransformation

All strains and plasmids used in this study are listed in Table S1 (Supporting Information). The plasmids used in this study are were constructed by circular polymerase extension cloning (CPEC)^[^ ^51^ ^]^ or Gibson assembly^[^ ^52^ ^]^ in *Escherichia coli* TransT1. The *ribABCDE* operon were amplified from the *E. coli* LS02T^[^ ^53^ ^]^ and *ribGBAH* operon were amplified from the *B. subtilis* CY46.^[^ ^54^ ^]^ Genes *omcA*, *mtrC, sulA, mreB, and ftsZ* were amplified from the *S. oneidensis* MR‐1 genome. All primers used in this study are listed in Table S2 (Supporting Information). All genes were then inserted into the appropriate sites of the plasmids (pHG12, pHG13), respectively. Finally, all plasmids to be electroformed into *S. oneidensis*.^[^ ^55^ ^]^

### Culture Condition and Flavin Measurements

*E. coli* strains were cultured in LB broth (10 g L^−1^ tryptone, 5 g L^−1^ yeast extract, 10 g L^−1^ NaCl) at 37 °C with 220 rpm. *S. oneidensis* MR‐1 were cultured at 30 °C in LB broth (50 mL) aerobically at 200 rpm for 12 h. When needed, culture media was supplemented with chloramphenicol (34 µg mL^−1^) and/or kanamycin (50 µg mL^−1^).

The culture supernatants were subjected to flavin measurements. All standard solutions and sample supernatants were filtered and assayed by a reverse‐phase C18 column (10 cm ×2.1 mm, 5 µm, Thermo‐Fisher Scientific, USA) using the Prominence UPLC system (Shimadzu, Japan), under the following conditions: mobile phase, 10 mm NaH_2_PO_4_, 30% methanol; flow rate, 0.8 mL min^−1^; peak detection at 445 nm (UV detector). The FAD, FMN, and RF reference standards were purchased from Sigma‐Aldrich (USA).

### Molecular Dynamics Simulations

All molecular simulations were carried out using the Gromacs2018.3 software.^[^ ^26^ ^]^ Structural displays of proteins and small molecules as shown in Figure S2 (Supporting Information). Then, imported proteins and small molecules structures was imported into AutoDock Tools 1.5.6^[^ ^56^ ^]^ for hydrogenation and charge treatment (Figure S3, Supporting Information). In this study, the configuration with the lowest binding energy was used as the optimal configuration for molecular dynamics simulations. Molecular dynamics simulation was carried out at 300 K for 80 ns with simulation time interval of 2 fs. The free energy of binding between receptor and ligand was calculated using MM‐PBSA.^[^ ^57^ ^]^

### MFC Measurements

Dual‐chamber MFCs (140 mL) with the electrodes connected via a 2 kΩ external resistor was used to record voltage output of every 30 min by data acquisition cards MPS‐110001 (Morpheus Electronics Technology Co. Ltd., China). Carbon cloth or carton felt (GasHub, Singapore) was used as the electrodes for the anode (1 ×1 cm^2^). Carbon cloth (GasHub, Singapore) was used as the electrodes for cathode (3 ×2.5 cm^2^) and the Nafion 117 membrane (GasHub, Singapore) was used to separate the anode and cathode and was pretreated as follows. The anolyte was M9 buffer (Na_2_HPO_4_, 6 g L^−1^; KH_2_PO_4_, 3 g L^−1^; NaCl, 0.5 g L^−1^; NH4Cl, 1 g L^−1^; MgSO_4_, 1 mm; CaCl_2_, 0.1 mm)), supplemented with 5% LB broth, 20 mm lactate with *S. oneidensis* MR‐1 in LB medium with a final concentration of OD_600_≈0.7. The catholyte was 50 mm K⁠_3_[Fe(CN)_6_], 50 mm KH_2_PO⁠_4_, and 50 mm K_2_HPO_4_. CV analysis with 1 mV s^−1^ scan rate was conducted on a three‐electrode configuration with an Ag/AgCl reference electrode. Linear sweep voltammetry (LSV) with a slow scan rate (0.1 mV s^−1^) was applied to obtain the polarization curves. The CV and LSV were controlled by CHI1010C (CHI instrument, Shanghai, China). Electrochemical impedance spectroscopy (EIS) experiments were measured using a multichannel potentiostat Solartron 1470E (Solartron Analytical, USA) over a frequency range of 10^−2^ to 10^5^ Hz with an amplitude of 10 mV.

### Electrode Preparation

The GO‐MWCNT composite solution was obtained by compounding 10% MWCNT dispersion with GO solution at 1:1 v/v ratio. The mixture underwent ultrasonic dispersion for 1 h, followed by stirring with magnetic agitator for 1 h. The CF/GO‐MWCNT composite electrode was prepared by soaking a piece of carbon felt (1 × 1 cm^2^) in GO‐MWCNT composite solution, which underwent ultrasonic treatment for 1 h and was then put into an oven to dry. RF was adsorbed onto the modified CF/GO‐MWCNT electrodes by electro‐aggregation.^[^ ^58^ ^]^

### SEM

To prepare the anodic biofilms for SEM, the carbon felt anodes were fixed overnight in 2.5% glutaraldehyde and then were washed three times with a 0.85% NaCl solution 10 min each wash. Next, the anodes were dehydrated with an ethanol series of 30%, 50%, 70%, 80%, and 90% ethanol 15 min each wash. The processed samples were dried overnight in a freeze dryer.

### Confocal Scanning Microscopy

Sample preparation for confocal imaging of the anodes was made as follows. Lightly cut off the anode carbon cloth, rinse with sterile water to remove the planktonic bacteria, and then stain with a red/green dye to 1:1 volume mixing, and then mix the measuring of the dye with electrode surface to biofilm dyed sample about ≈15–20 min, the samples with tin foil cover the light stand for dyeing 15 min. The scanning process was advanced layer by layer from the surface of the biofilm to the electrode interface. Two laser channels, red and green, were selected. The excitation light was green channel 480 nm and red channel 490 nm, while the detection light was green channel 500 nm and red channel 635 nm.

### Biomass Measurements

The biomass attached to the anode was analyzed based on the protein concentration as measured by a BCA protein assay kit (Beyotime Co., China).

### Spectral Analysis

Fourier transform infrared (FTIR) spectroscopy was carried out with an FTIR spectrometer (Nicolet iS20, Thermo‐Fisher Scientific, USA]). Before tablet pressing, all samples and the KBr were dried, and the sample was mixed with KBr (1:100, v/v). X‐ray photoelectron spectroscopy measurements were made using monochrome Al K*α* radiation (K‐Alpha, Thermo‐Fisher Scientific, USA).

## Conflict of Interest

The authors declare no conflict of interest.

## Supporting information

Supporting Information

## Data Availability

The data that support the findings of this study are available from the corresponding author upon reasonable request.
