# Supplementary material for: Elongated Riboflavin‐Producing Shewanella o neidensis in a Hybrid Biofilm Boosts Extracellular Electron Transfer
Source: Adv Sci (Weinh). 2023 Jan 29;10(9):2206622. doi: 10.1002/advs.202206622 (PMC10037984; doi:10.1002/advs.202206622)
Supplement: Supplementary file 1 — Supporting Information [file ADVS-10-2206622-s001.pdf]

ADVANCED SCIENCE
Open Access

Supporting Information

for *Adv. Sci.*, DOI 10.1002/advs.202206622

Elongated Riboflavin-Producing *Shewanella oneidensis* in a Hybrid Biofilm Boosts Extracellular Electron Transfer

*Juntao Zhao, Feng Li, Shutian Kong, Tao Chen, Hao Song and Zhiwen Wang**

Supplementary Material for

Elongated riboflavin-producing *Shewanella oneidensis* in a hybrid biofilm boosts extracellular electron transfer

Juntao Zhao[1, 2, 3 †], Feng Li[1, 2, 3 †], Shutian Kong[1, 2, 3], Tao Chen[1, 2, 3], Hao Song[1, 2, 3], Zhiwen Wang[1, 2, 3 *]

[1]*Frontier Science Center for Synthetic Biology, Tianjin University, Tianjin 300072, People's Republic of China*

[2]*Key Laboratory of Systems Bioengineering (Ministry of Education), Tianjin University, Tianjin 300072, People's Republic of China*

[3]*School of Chemical Engineering and Technology, Tianjin University, Tianjin 300072, People's Republic of China*

† These authors contributed equally to this work.

* Corresponding author: Zhiwen Wang

E-mail address: zww@tju.edu.cn (Zhiwen Wang)

Supplementary Text

Plasmid construction and electroransformation

The plasmids used in this study are constructed by circular polymerase extension cloning (CPEC) or Gibson assembly in *Escherichia coli* TransT1 (Table S1). In short, the overlapping DNA fragments with 15-25 bp were amplified by PCR, and then assembled into circular plasmids by CPEC or Gibson, and the resulting plasmids was used to identify by sequencing analysis. The primers used are shown in Table S2. Correct plasmids were transformed into TransT1 and filter the correct transformant by colony PCR. The gene sequence (*ribABCDE* and *ribGBAH*) amplified were inserted into the corresponding sites of the plasmids carrying the promoter Ptac, Ptrc, Pj23119, Pbad, Ptet, and Lacuv5, respectively, resulting in 12 expression vectors. The *omcA* and *mtrC,* were amplified from the *S. oneidensis* MR-1 genome which were then inserted into the corresponding sites of the plasmids carrying the promoter Llaco-1 respectively, resulting in 2 expression vectors. *sulA, ftsZ* and *mreB* were amplified from the *S. oneidensis* MR-1 genome which were then inserted into the corresponding sites of the plasmids carrying the promoter lacuv5, resulting in three expression vectors. All plasmids were electroformed into *S. oneidensis*.

For electroporation, 5 μL of the correct plasmids were added into 50 μL competent cells and mixed, and then electroporation was carried out in a 0.1 cm tube at room temperature. After applying a pulse (setting :10 μF, 600 Ω, 1.2 kV on a Bio Rad micropulser TM), 1 mL LB liquid medium was added and transferred to a 2 mL Eppendorf tube. Cells were resuscitated at 30°C, 200 rpm for 2 h, and then

centrifugally coated onto agar plates containing antibiotics. The plates were incubated at 30°C until colonies appeared.

Culture condition and flavin measurements

Overnight *S. oneidensis* MR-1 (pHG12 vector containing the flavin synthesis genes or Mtr-related genes or cell morphology-related genes) suspension was transferred to Luria-Bertani (LB) medium (the ratio was 1:100 (v/v)) with 50 μg mL^{-1} kanamycin and different concentration inducer, respectively. Overnight *S. oneidensis* MR-1 (pHG13 vector containing the flavin synthesis genes or cell morphology-related genes) suspension was transferred to LB medium (the ratio was 1:100 (v/v)) with 34 μg ml^{-1} chloramphenicol and different concentration inducer, respectively. The ptac, pLlacO1, and lacUV5 promoters was induced at IPTG concentrations of 1 mM, respectively. Pbad promoter was induced at an arabinose concentration of about 10 mM in *S. oneidensis* MR-1 (pHG13 vector containing the flavin synthesis genes), 3 mM in *S. oneidensis* MR-1 expressed cell morphology-related genes. The ptet promoter was induced at an aTc concentration of about 1000 ng ml^{-1}. After growing 12 h at 30°C with 200 rpm, the culture broth was subjected to flavin measurements.

Molecular dynamics simulations

The crystal water of cytochrome MtrC, OmcA (PDB ID: 4LM8, 4LMH) was removed by PyMOL 2.5.2 software.[1] Chem3D 18.0 software[2] was used to minimize energy for RF and FMN (PubChem database). In this study, the most probable binding sites for RF and FMN were first identified and the 50 highest-scoring conformation models was selected by flexible docking method. The configuration with the lowest

binding energy was used as the optimal configuration for molecular dynamics simulations. The simulation temperature was 300 K, and the system was optimized by using the fastest descent method for 50000 steps. Then, the optimized system was balanced by NVT and NPT ensemble with step length of 2 fs and total time of 100 Ps, and the system position was restricted during the balancing process. Finally, molecular dynamics simulation was carried out at 300 K for 80 ns with simulation time interval of 2 fs. The free energy of binding between receptor and ligand was calculated using MM-PBSA. Visualization was performed using Pymol 2.4.1.

MFC measurements

Overnight *S. oneidensis* MR-1 harboring plasmid culture suspensions were inoculated into 100 ml fresh LB broth with corresponding antibiotics at 30°C with 200 rpm under the control of the inducible promoter. The medium was supplemented with 0.1 mM IPTG for promoter Lacuv5 and 0.25, 0.5, 0.75 and 1 mM IPTG for promoter LIaco-1, 1000 ng ml^{-1} aTc for promoter Ptet, or 10 mM arabinose for promoter Pbad. After around 12 h culture, the concentrations of cell suspensions were adjusted to the same level (OD_{600}≈0.7), and the suspensions were dispersed into the anode chambers of MFC including M9 buffer (3 g l^{-1} KH_2PO_4, 6 g l^{-1} K_2HPO_4, 0.5 g l^{-1} NaCl and 1g l^{-1} NH_4Cl), 1 ml 1 M $MgSO_4$, 1 ml 0.1 M $CaCl_2$, 1 ml 5% (v/v) LB broth, 20 mM lactate, kanamycin or chloramphenicol and different inducer) of three H cell reactors for parallel, with a working volume of 140 ml. The anodes were purged with nitrogen gas to exclude oxygen. The cathode chambers of MFC was supported 50 mM $K_3[Fe(CN)_6]$, 50 mM KH_2PO_4 and 50 mM K_2HPO_4. A 2 kΩ external resistor was

connected to both anode and cathode. MFC reactors were incubated at 30℃, and the voltage outputs were continuously recorded by data acquisition cards MPS-110001 (Morpheus Electronics Technology Co. Ltd., China).

Carbon cloth or carbon felt (Gashub, Singapore) was used as the anode (1 cm×1 cm), which was pretreated as follows. First, soak with 1 M hydrochloric acid, then soak with acetone, clean with deionized water, dry and reserve. The Nafion 117 membrane (GasHub, Singapore) is used to separate the anode and cathode and is pretreated as follows. First, wash them with distilled water several times. Then soak with 1 M hydrochloric acid, clean with sterile water for many times, and store in sterile distilled water.

Electrode preparation and characterization

The preparation method of CF/GO-MWNT/PRF composite electrode is described above.[3] The CF/GO-MWCNT electrode (1×1 cm) was first electrochemically polished in 0.1 M KCl solution, and the scanning was repeated at a scanning rate of 0.1 V/s at least 15 times between −1.0 and +1.0 V until a constant cyclic voltammetry (CV) was obtained. Subsequently, the polished electrode was successively rinsed with ultrapure water and transferred into 0.1 M phosphate buffered saline (PBS, pH 7.0) solution containing 0.15 M NaCl and 1 mM RF. At a scanning speed of 50 mV/s, the electric polymerization of riboflavin was obtained by repeated scanning 30 times in a potential window of −1.0 ~ +1.5 V. Finally, the functionalized electrode (CF/GO-MWCNT/PRF) was rinsed with ultrapure water for three times and then dried at 60°C overnight.

Electrode attached biomass measurement

To quantify the attached biomass on electrodes, the total protein concentration on the electrodes was determined. Anode were removed from the electrochemical cells, washed twice in PBS buffer, and incubated in 3 mL of 0.2 M NaOH for 10 min at 95°C to solubilize the attached cells. The supernatant was analyzed using a BCA protein assay kit (Beyotime Co., China) according to the manufacturer's instructions.

Table S1 Strains and Plasmids used in this study

Strains or Plasmids	Feature(s)	Source
E. coli Trans T1	Conventional clone host bacteria	Our lab
S. oneidensis MR-1	Parent strain	Our lab
Ptac-5	Carrying pHG12-Ptac-ribABDEC	This study
Ptac-Ptrc-5	Carrying pHG12-Ptac-ribABD-Ptrc-ribEC	This study
Ptrc-Ptrc-5	Carrying pHG12-Ptrc-ribABD-Ptrc-ribEC	This study
Lacuv5-5	Carrying pHG13-Lacuv5-ribABDEC	This study
Ptet-5	Carrying pHG13-Ptet-ribABDEC	This study
Pbad-5	Carrying pHG13-Pbad-ribABDEC	This study
Ptac-4	Carrying pHG12-Ptac-ribGB-P43-ribAH	This study
Pj23119-4	Carrying pHG12-Pj23119-ribGB-P43-ribAH	This study
Pj23119-Pj23119-4	Carrying pHG12-Pj23119-ribGB-Pj23119-ribAH	This study
Lacuv5-4	Carrying pHG13-PLacuv5-ribGB-P43-ribAH	This study
Ptet-4	Carrying pHG13-Ptet-ribGB-P43-ribAH	This study
Pbad-4	Carrying pHG13-Pbad-ribGB-P43-ribAH	This study
Ptet-5/POmcA	Carrying pHG13-Ptet-ribABDEC, pHG12-LIaco-1-OmcA	This study
Ptet-5/PMtrC	Carrying pHG13-Ptet-ribABDEC, pHG12-LIaco-1-MtrC	This study
Ptet5-sulA	Carrying pHG13-Ptet-ribABDEC-Lacuv5-sulA	This study
Ptet-5-mreB	Carrying pHG13-Ptet-ribABDEC-Lacuv5-mreB	This study
Ptet-5-ftsZ	Carrying pHG13-Ptet-ribABDEC-Lacuv5-ftsZ	This study
Ptet5/PMtrC-sulA	Carrying pHG13-Ptet-ribABDEC, pHG12-LIaco-1-MtrC-Pbad-sulA	This study
pHG12	reppBBR1， Km^r	Our lab
pHG13	repColE， Cm^R	Our lab

pHG12-Ptac-ribABDEC	with the *ribA*, *ribB*, *ribC*, *ribD* and *ribE*	This study
pHG12-Ptac-ribABD-Ptrc-ribEC		This study
pHG12-Ptrc-ribABD-Ptrc-ribEC		This study
pHG13-Lacuv5-ribABDEC		This study
pHG13-Ptet-ribABDEC		This study
pHG13-Pbad-ribABDEC		This study
pHG12-Ptac-ribGB-P43-ribAH		This study
pHG12-Pj23119-ribGB-P43-ribAH		This study
pHG12-Pj23119-ribGB-Pj23119-ribAH		This study
pHG13-Lacuv5-ribGB-P43-ribAH	with the *ribG, ribB, ribA* and *ribH* gene inserted	This study
pHG13-Ptet-ribGB-P43-ribAH		This study
pHG13-Pbad-ribGB-P43-ribAH		This study
pHG12-LIaco-1-omcA	Plasmid with the *omcA* gene inserted	This study
pHG12-LIaco-1-MtrC	Plasmid with the *mtrC* gene inserted	This study
pHG13-ptet-ribABDEC-Lacuv5-sulA	Plasmid with the *ribA*, *ribB, ribC, ribD, ribE* and *sulA* gene inserted	This study
pHG13-ptet-ribABDEC-Lacuv5-mreB	Plasmid with the *ribA*, *ribB, ribC, ribD, ribE* and *mreB* gene inserted	This study
pHG13-ptet-ribABDEC-Lacuv5-ftsZ	Plasmid with the *ribA*, *ribB, ribC, ribD, ribE* and *ftsZ* gene inserted	This study
pHG12-LIaco-1-MtrC-Pbad-sulA	Plasmid with *mtrC* and *sulA* gene inserted	This study

Table S2 Primer used in this study

Primers	Sequences (5’→3’)
ribGBAH-1	atggaagagtattatatgaagct
ribGBAH-2	caggcggccgctactagtaattaacggatacgaataggtgatc
PHG12-1	agcttcatataatactcttccatcatatgctatggtccttgttggtg
PHG12-2	gttaattactagtagcggccgcctgca
PHG12-3	agcttcatataatactcttccatcatatgctatggtccttgttggtg
PHG12-4	gttaattactagtagcggccgcctgca
ribGBAH-3	ctagtaataattttgtttaactttaagaaggagatataagaggaagatttgcatgtttcatccg
ribGBAH-4	taaagttaaacaaaattattactagtattatacctaggactgagctagctgtcaactaaaagccgttttcgcttaag
ribGBAH-5	atggaagagtattatatgaagc
ribGBAH-6	accgctcatttgaataagatatctcgagca
PHG13-1	gaataagatatctcgagcaaataaaacgaaaggctcagtcgaaag
PHG13-2	gcttcatataatactcttccatggatcctgtatatctccttctta
PHG13-3	cttcatataatactcttccatgctagctgtatatctccttcttaaagt
PHG13-4	aatactcttccatggatcctgtatatctccttcttaaagttaaac
ribABCDE-1	aattgtgagcggataacaatttgtggtcacacagaattcgag
ribABCDE-2	aggcggccgctactagttcaggcttctgtgcctggttgatt
PHG12-7	actagtagcggccgcctgcagg
PHG12-8	agcttcatataatactcttccatcatatgctatggtccttgttggtg
ribABCDE-3	ttgacaattaatcatccggctcgtataatggtcgacacatacgtcaaacgc
ribABCDE-4	cattatacgagccggatgattaattgtcaatcatgcacccactaaatgcag
ribABCDE-5	ttgacaattaatcatccggctcgtataatgtgtggtcacacagaattcgag
ribABCDE-6	cattatacgagccggatgattaattgtcaatctagaagcggccgcgaattcg
ribABCDE-7	aataattttgtttaactttaa
ribABCDE-8	atttgctcgactcgagtcaggcttctgtgcctggtt
PHG13-5	cacagaagcctgactcgagtcgagcaaataaaa
PHG13-6	cttaaagttaaacaaaattattcctaggaattgttatccgctca
PHG13-7	gagctcgaattctgtgtgaccacatatatctccttcttaaagttaaacaaaa
PHG13-8	ttcttaaagttaaacaaaattattcctaggcttttctctatcactga
OmcA-F	ttaagaaggagatatacatatgatgatgaaacggttcaatttc
OmcA-R	ttgctcgagctgctagcttattagttaccgtgtgcttcca
MtrC-F	ctttaagaaggagatatacatatgatgatgaacgcacaaaaatc
MtrC-R	agcctttcgttttatttgctcgagttacattttcactttagtgtga
PHG12-L-1	catagttatatctccttcttaaagt

PHG12-L-2	taagctagcagctcgagcaaat
sulA-1	taactttaagaaggagatatacatatgaacaaactattaggtaatg
sulA-2	ttatttgctcgagccgctagcttaatgaacagagctgaaaaaagc
mreB-1	ctttaagaaggagatatacatgtgtttacttggctaaaggggtt
mreB-2	atttgctcgagccgctagcttatcccttcttaatcataagtt
ftsZ-1	tttaagaaggagatatacatatgtttgagatcatggacactca
ftsZ-2	atttgctcgagccgctagcttagtcagcttgcttacgcaa
PHG13-F	cgctctcctgagtaggacaaatagcggccgctgcagcgttcggct
PHG13-R	actagtaatttgtcctactcaggaga
Arac-sulA-F	gctttcgaacgttaccaattatgacaacttg
Arac-sulA-R	cgggactctggggatttgtcctactcaggagagcg
PHG12-MtrC-F	taggacaaatccccagagtcccgctcagaagaac
PHG12-MtrC-R	ataattggtaacgttcgaaagcaaattcgacccgg

Table S3 Single factor test of exogenous addition of flavins

	Vessel number											
Flavins (μmol/l)	1	2	3	4	5	6	7	8	9	10	11	12
RF	20	40	60	80	-	-	-	-	-	-	-	-
FMN	-	-	-	-	20	40	60	80	-	-	-	-
FAD	-	-	-	-	-	-	-	-	20	40	60	80

Table S4 Multivariate test factors of exogenous addition flavins

Vessel number	Flavins (40 μmol l^{-1}) (nRF:nFMN:nFAD)
1	1:5:0
2	1:2:0
3	1:1:0
4	2:1:0
5	5:1:0
6	2:3:1
7	3:2:1
8	1:1:1

Table S5 Parameters associated with the docking of RF and FMN to the outer membrane cytochromes OmcA and MtrC

OMC	Position	Heme	RF		FMN	
			Affinity (kcal/mol)	RF-heme distance (Å)	Affinity (kcal/mol)	FMN-heme distance (Å)
OmcA	1	Heme5	-7.4	7.9	-7.1	8.1
	2	Heme7	-6.7	11.2	-6.1	6.0
MtrC	1	Heme7	-6.4	6.7	-7.3	6.2
	2	Heme9	-6.8	6.8	-6.3	6.7

Table S6 Binding free energies (kJ mol^{-1}) with RF/FMN and OmcA/MtrC

Binding free energy（kJ/mol)	RF	FMN
OmcA	-105.875	-128.779
MtrC	-150.667	-122.841

Table S7 Riboflavin synthesis pathway of different strains and the corresponding enzymes in the pathway.

Shewanella		***Escherichia coli***		***Bacillus subtilis***	
Gene	Protein	Gene	Protein	Gene	Protein
ribA/ribAB	GTP cyclization hydrolase	*ribA*	GTP cyclization hydrolase II	*ribA*	GTP cyclization hydrolase II
ribD	ArPP phosphatase	*ribD*	Pyrimidine deaminase/reductase	*ribD*	Pyrimidine deaminase/reductase
ribE	DRL synthase Riboflavin synthase	*ribH*	DRL synthase	*ribE*	Riboflavin synthase
ribC	Riboflavin synthase	*ribC*	Riboflavin synthase	*ribH*	DRL synthase
ribB/ribBA	DHPB synthase	*ribB*	DHPB synthase	*ribA*	DHPB synthase
ribF	FMN adenosine transferase RF kinase	*ribF*	FMN adenosine transferase RF kinase	*ribC*	FMN adenosine transferase RF kinase

Table S8 Summary of the reported titer of flavins with engineered *Shewanella*

Strain	Medium	Flavins	Strategies	Ref
S. oneidensis MR-1	LB	39.7 μM	Overexpressing gene cluster *ribADEHC* from *B. subtilis* was assembled under the control of promoters Ptet	[4]
S. oneidensis MR-1	Mineral medium	1.33 μM	Gene cluster *ribDBAE* were coexpressed	[5]
S. oneidensis MR-1	basal medium	26.15 μM	Gene cluster *ribADEHC* from *B. subtilis* was assembled under the control of promoters Ptac	[6]
Shewanella sp. HN-41	LB	0.45 μM	Natural secretion	[7]
Shewanella sp. RQs-106	LB	6.04 μM	Immobilized AQS (iAQS)	[8]
S. oneidensis MR-1	M4 minimal medium	4.17 μM	Overexpression of the gene encoding the putrescine decarboxylase *speC*	[9]
S. oneidensis MR-1	LB	889.3 μM	Gene cluster *ribABCDE* from *E. coli* was assembled under the control of promoters Ptet	This study

Table S9 The comparison of the power density of MFCs with different strategies in *Shewanella*

Strategy	Microorganism	Power density (mW/m^2)	Ref
Intracellular reformation	*Shewanella oneidensis* MR-1	0.13	[5]
	Shewanella oneidensis MR-1	233	[6]
	Shewanella oneidensis MR-1	1120	[4]
	Shewanella oneidensis MR-1	37	[10]
	Shewanella oneidensis MR-1	167	[11]
Materials engineering	*Shewanella oneidensis* MR-1	147.9	[12]
	Shewanella oneidensis MR-1	3210	[13]
	Shewanella oneidensis MR-1	3580	[14]
	Shewanella oneidensis MR-1	707	[3]
	Shewanella oneidensis MR-1	843	[15]
	Shewanella oneidensis MR-1	1460	[16]
	Shewanella putrefaciens	257	[17]
	Shewanella oneidensis MR-1	1459	[18]
	Shewanella putrefaciens	1280	[19]
	Shewanella putrefaciens	1137	[20]
	Shewanella oneidensis MR-1	1326	[21]
	Shewanella putrefaciens	3632	[22]
	Escherichia coli	3444	[23]
	Shewanella oneidensis MR-1	3736	This study

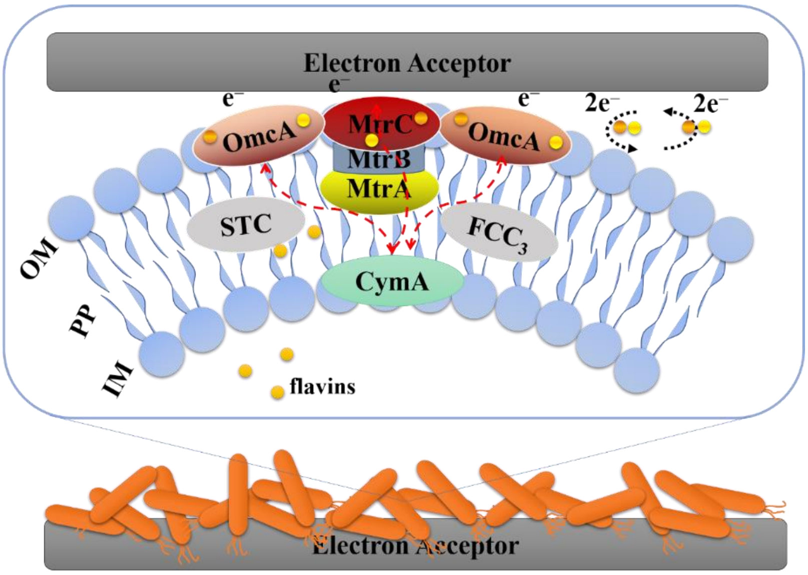

Fig. S1 Schematic illustration of the bidirectional extracellular electron transfer (EET) models of *Shewanella oneidensis*.

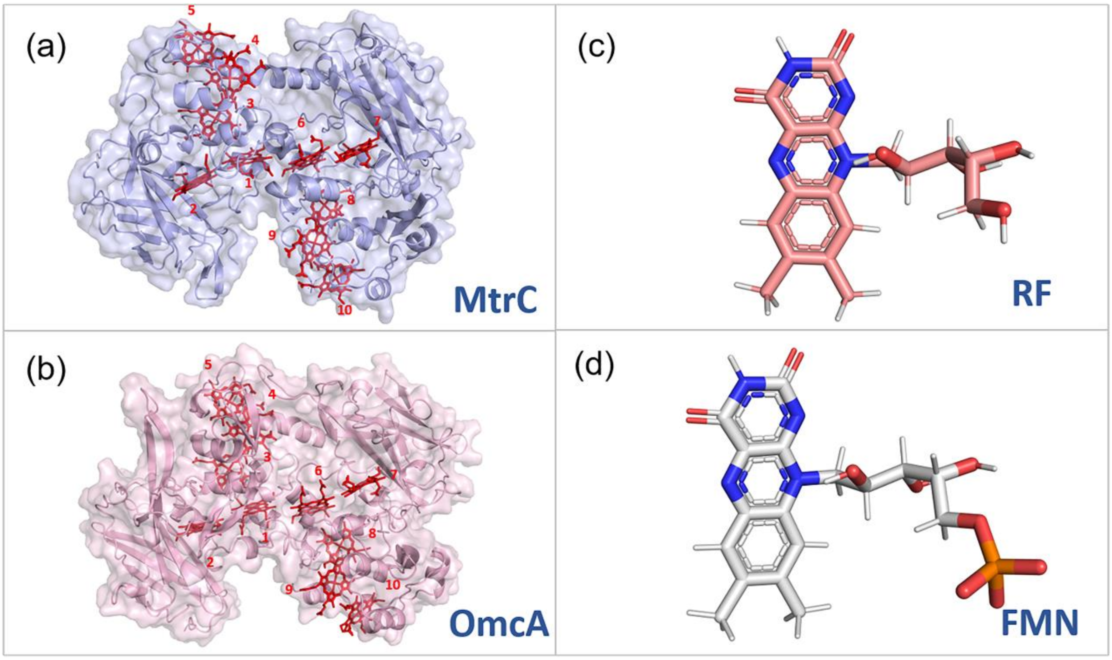

Fig. S2 Structure display of proteins and small molecules. (a) Crystal structure of MtrC (PDB ID: 4LM8). (b) Crystal structure of OmcA (PDB ID: 4LMH), the red sticks represent the structure of heme. (c) The structure of RF molecules (d) The structure of FMN molecules.

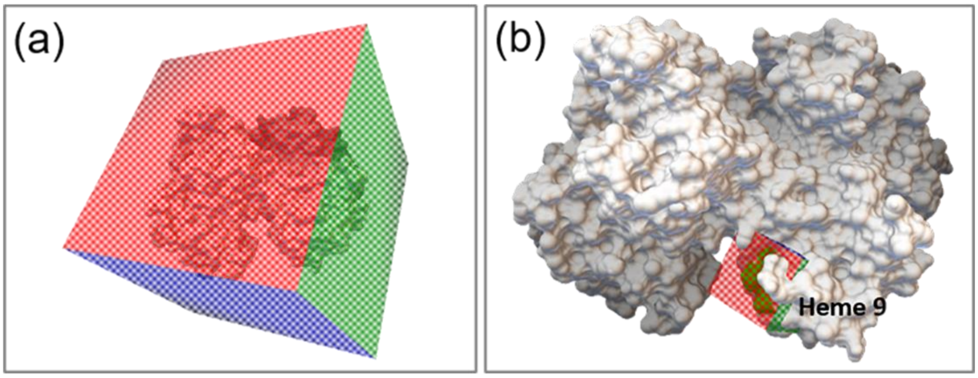

Fig. S3 Main aspects in molecular docking through AutoDock. (a) Grid map includes the whole protein molecule and initial docking simulation. (b) Refined docking simulation with grid map located at the in the nearest heme, the searching space was centered on heme 9 with the size of 40 X 40 X 40Å.

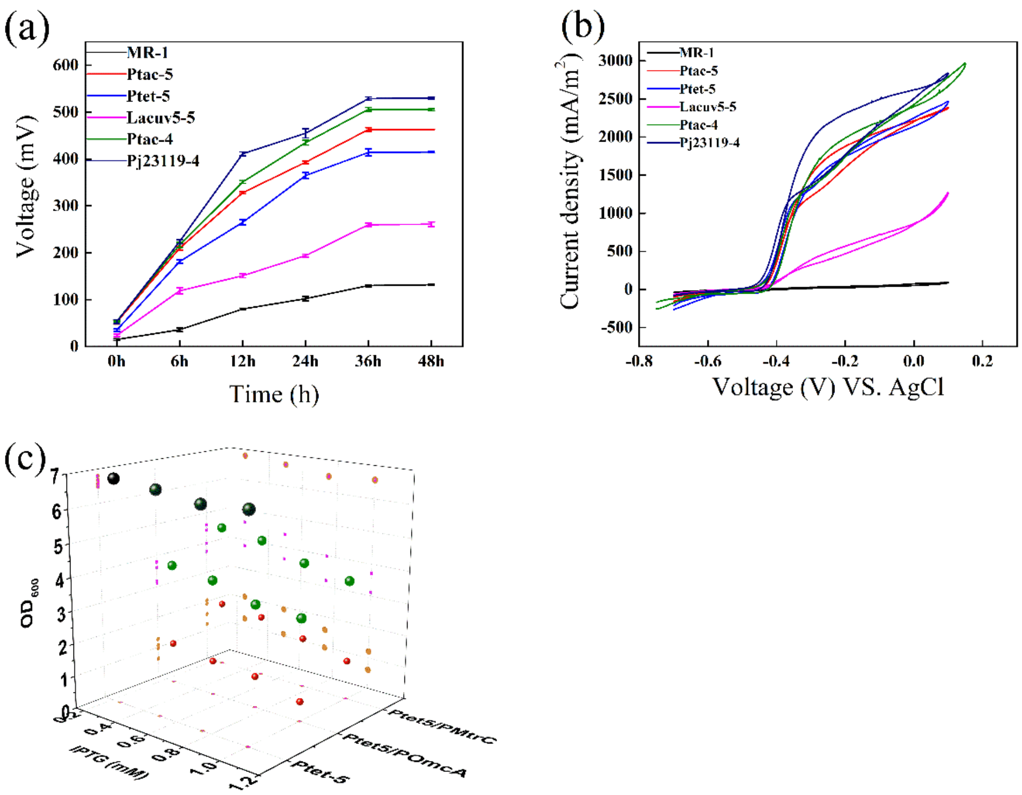

Fig. S4 Bioelectrochemical characterization of engineered *Shewanella* strains with different levels RF production. (a) Voltage output in MFCs. (b) Cyclic voltammetry (CV) curves. (c) Growth curve of engineered strain overexpressing cytochrome proteins. The red sphere represents the growth of the engineering strain induced by IPTG at different concentrations when OD=0.4, the green sphere represents the growth of the engineering strain induced by IPTG at different concentrations when OD=4, and the black sphere represents the growth of the control strain induced by IPTG at different concentrations. The error bar represents the standard deviation of three independent experiments.

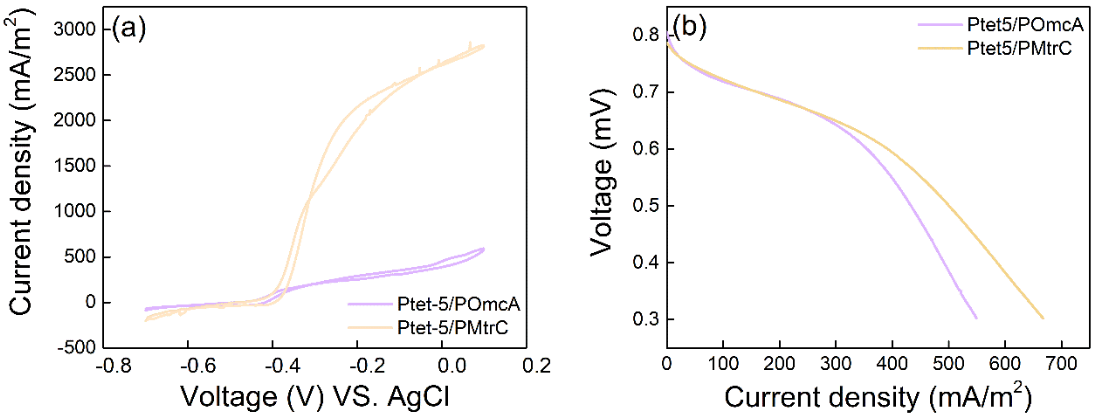

Fig. S5 Bioelectrochemical characterization of engineered *Shewanella* strains with overexpressing cytochrome proteins at 0.5 mM induction concentration. (a) Cyclic voltammetry curves. (b) MFC polarization curves.

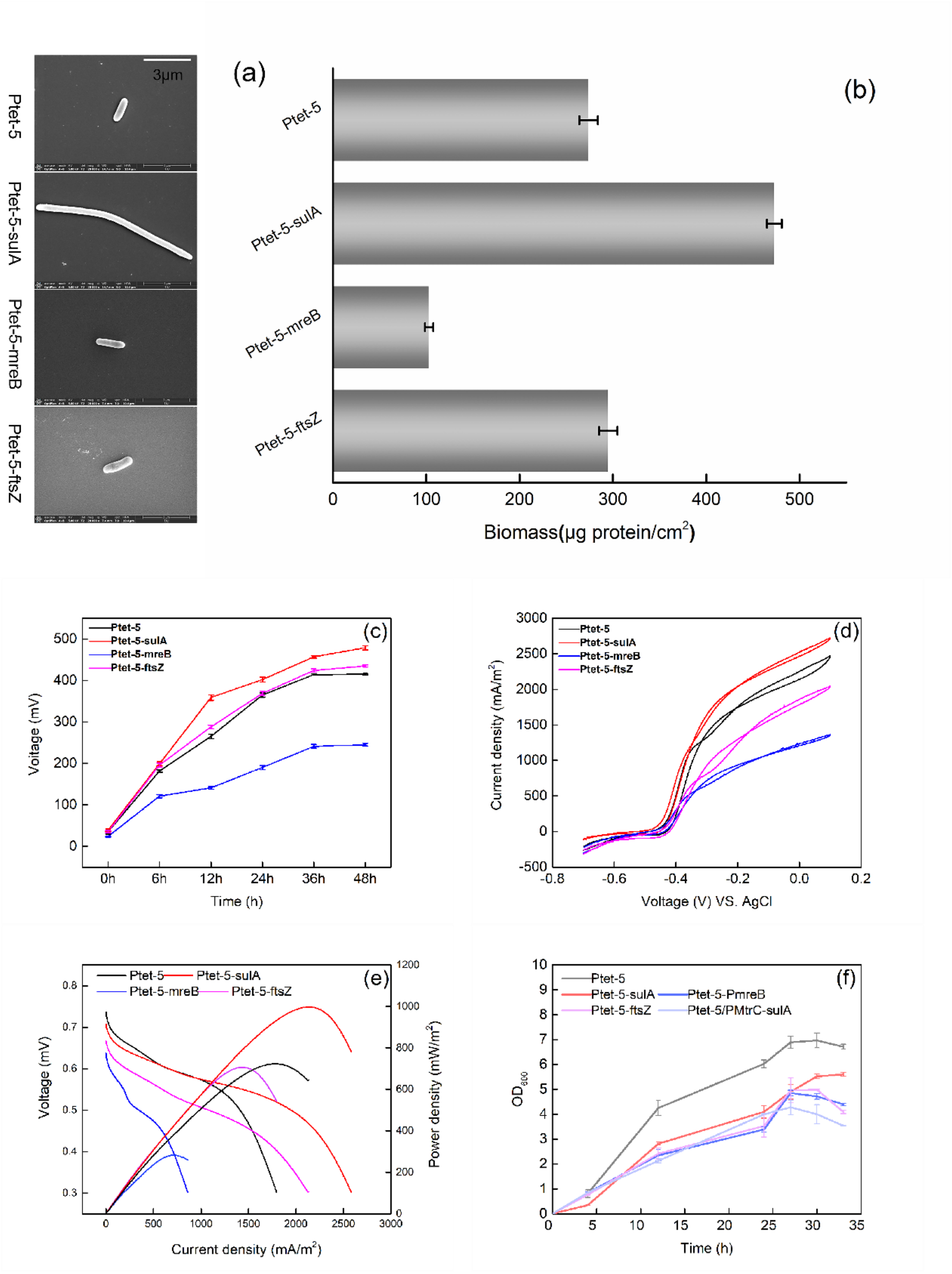

Fig. S6 The effects of different cell morphology-related genes (e.g., *sulA*, *mreB* and *ftsZ*) on cell morphology, biofilms and EET. (a) SEM of the overexpressing cell morphology-related genes engineered strains. (b) The biomass of overexpression of the cell morphology-related genes engineered strains and control strain of biofilm on electrode surface. (c-e) Bioelectrochemical characterization of engineered strains overexpressed of the cell morphology-related genes and control strain. (f) Growth curve of engineered strains overexpressing morphology-related genes. The error bars represent the standard deviation from three independent experiments.

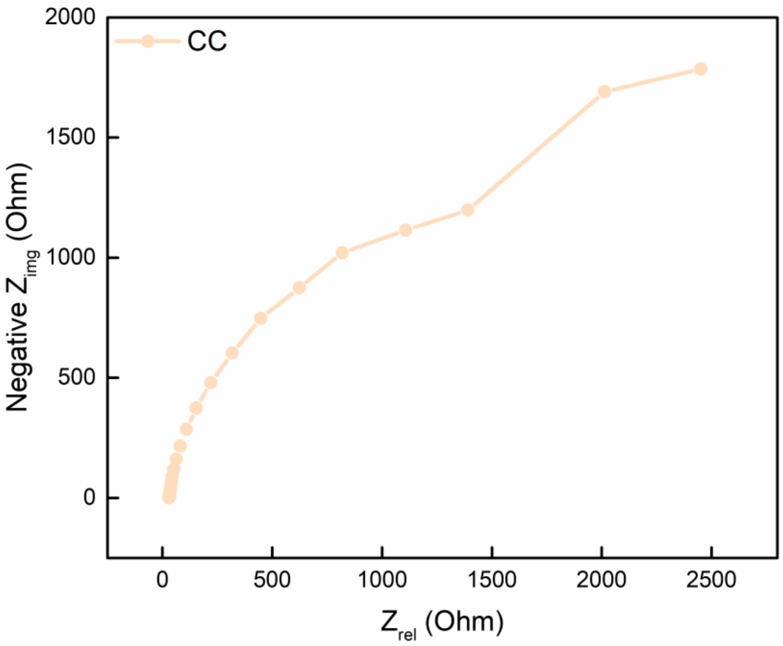

Fig. S7 Nyquist plots of electrochemical impedance spectroscopy scanned at open circuit potential over 0.01−100 000 Hz with a 10-mV perturbation.

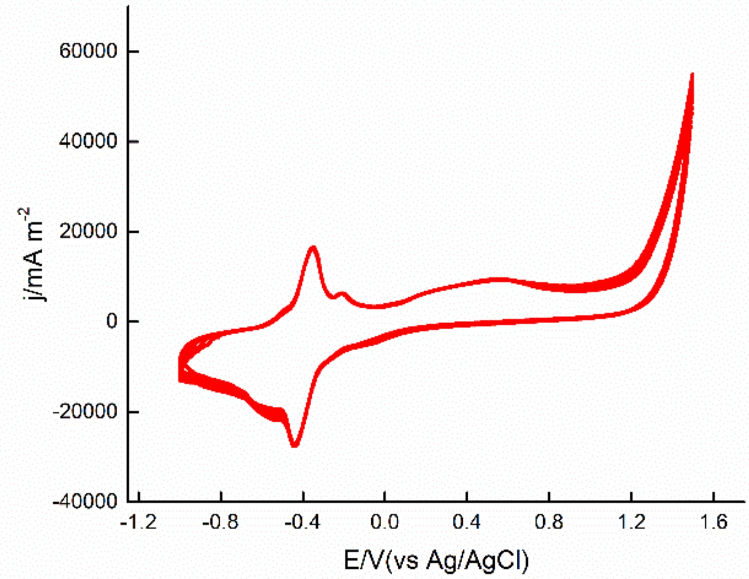

Fig. S8 Electropolymerization CV at a scanning speed of 50 mV/s for 1 mM riboflavin in 0.1 M PBS solution with the addition of 0.15 M NaCl (pH 7.0).

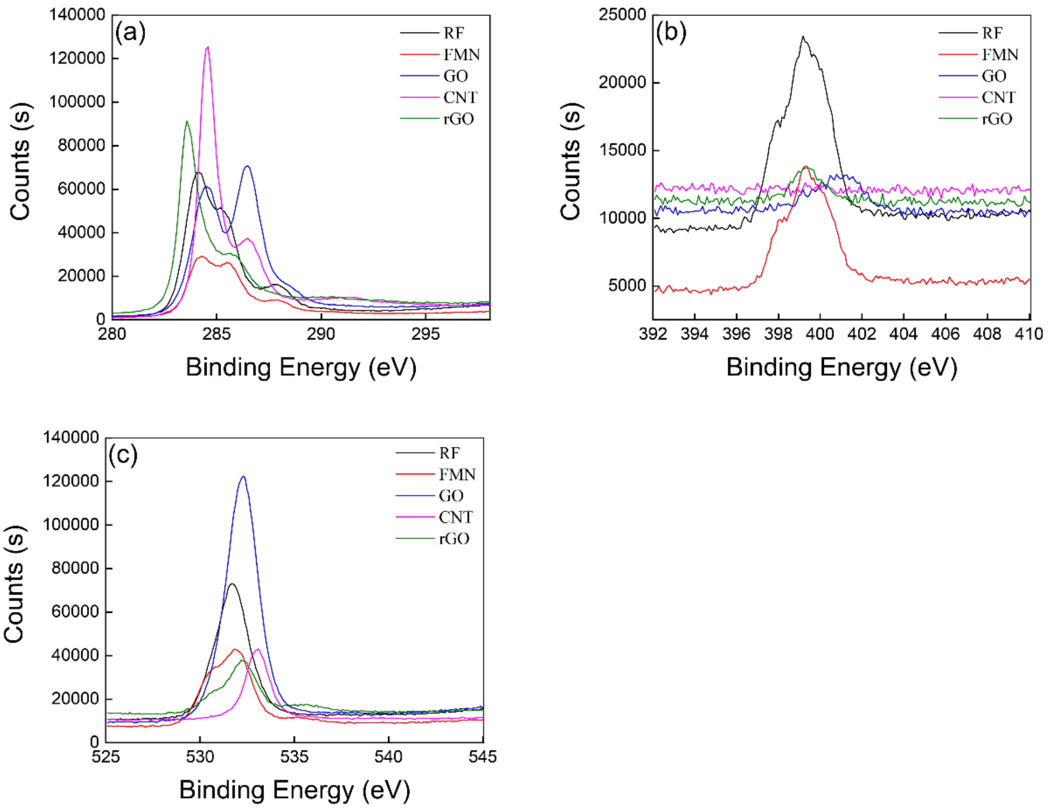

Fig. S9 Spectroscopic characterizations of the modified electrodes. (a) The C1s XPS spectra of the modified electrodes. (b) The N1s XPS spectra of the modified electrodes. (c) The O1s XPS spectra of the modified electrodes.

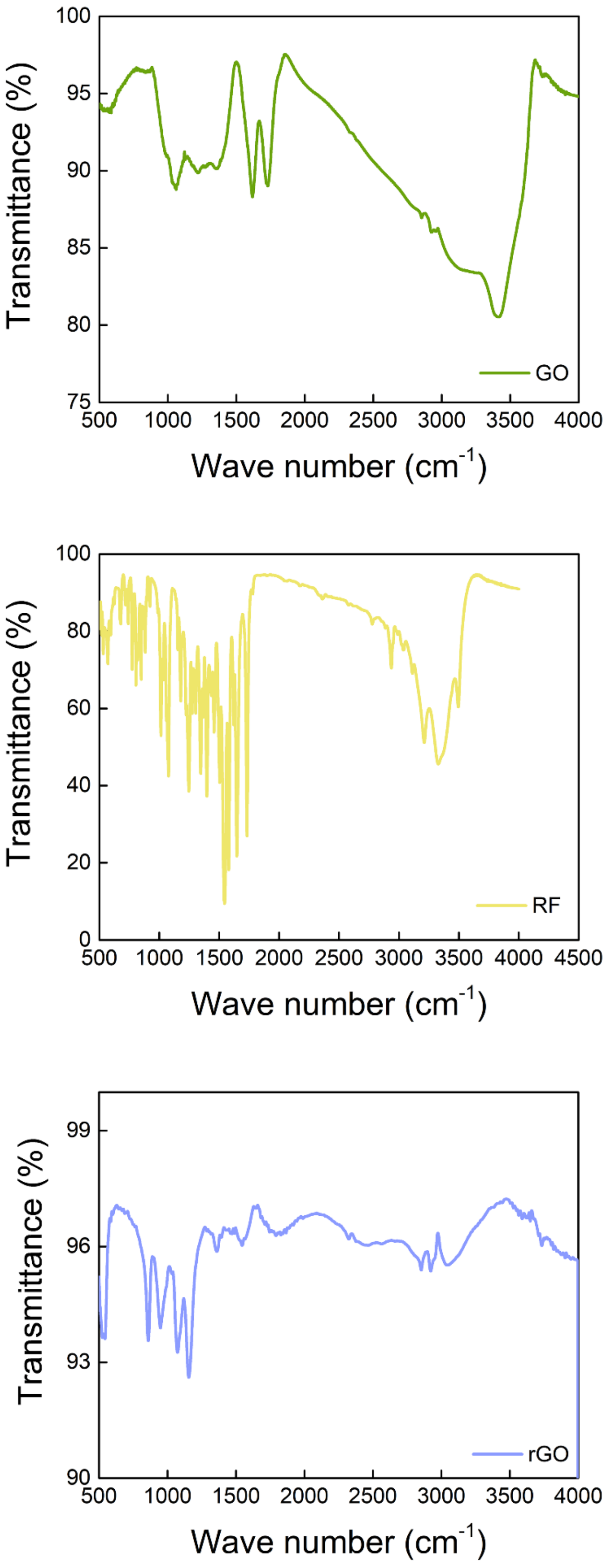

Fig. S10 FTIR spectra of the modified electrodes.

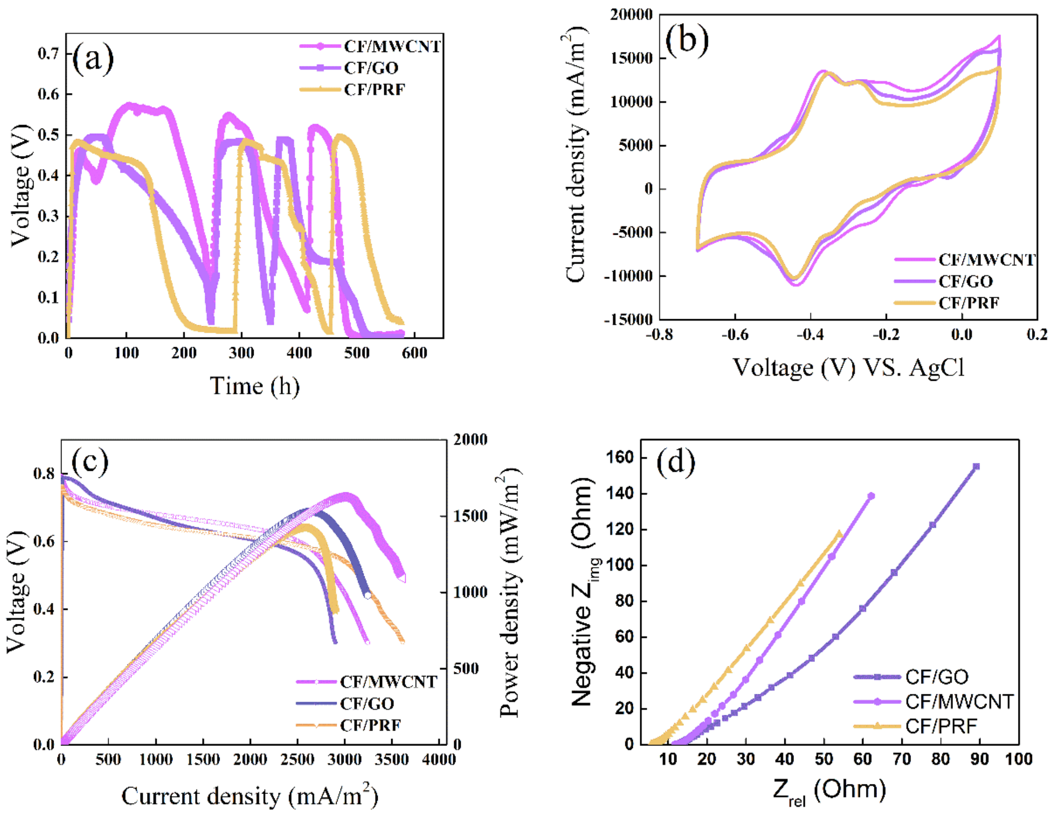

Fig. S11 Performance comparison of *Shewanella* MFCs with different anodes. (a) Voltage output of MFCs with different anodic materials. (b) CV curves of MFCs with different anodic materials. (c) I-V curves (left axis) and power polarization (right axis) curves of the MFCs with five different anode materials. (d) Nyquist plots of electrochemical impedance spectroscopy scanned at open circuit potential over 0.01−100000 Hz with a 10-mV perturbation. The error bars represent the standard deviation from three independent experiments.

References

[1] X. Robert, P. Gouet. *Nucleic Acids Res.* **2014,** 42, W320-W324.

[2] S. Ayers, T. N. Graf, A. F. Adcock, D. J. Kroll, Q. Shen, S. M. Swanson, S. Matthew, E. J. C. de Blanco, M. C. Wani, B. A. Darveaux, C. J. Pearce, N. H. Oberlies, *J. Antibiot.* ***2012,*** 65, 3-8.

[3] L. Zou, X. Wu, Y. Huang, H. Ni, Z.-e. Long, *Front. Microbiol.* **2019**, 9 (3293).

[4] T. Lin, W. Ding, L. Sun, L. Wang, C.-G. Liu, H. Song, *Nano Energy* **2018,** 50, 639.

[5] D. Min, L. Cheng, F. Zhang, X. N. Huang, D. B. Li, D. F. Liu, T. C. Lau, Y. Mu, H. Q. Yu, *EST* **2017**, 51 (9), 5082.

[6] Y. Yang, Y. Ding, Y. Hu, B. Cao, S. A. Rice, S. Kjelleberg, H. Song, *ACS Synth. Biol.* **2015**, 4 (7), 815.

[7] D. Wu, D. Xing, X. Mei, B. Liu, C. Guo, N. Ren. *J. Hydrog. Energy,* **2013**, 38, 15568-15573.

[8] H. Lu, Y. Zhou, Z. Fu, X. Wang, J. Zhou, W. Guo. *J. Haz. Mat.* **2022**, 431, 128595.

[9] M. Edel, G. Sturm, K. Sturm-Richter, M. Wagner, J. N. Ducassou, Y. Coute, H. Horn, J. Gescher. *Biotechnol. Biofuels* **2021**, 14.

[10]A. Vellingiri, Y. E. Song, G. Munussami, C. Kim, C. Park, B. H. Jeon, S. G. Lee, J. R. Kim. *J. Chem. Technol. Biotechnol*. **2019**, 94, 2115-2122.

[11] T. Liu, Y. Y. Yu, X. P. Deng, C. K. Ng, B. Cao, J. Y. Wang, S. A. Rice, S. Kjelleberg, H. Song, *Biotechnol. Bioeng.* **2015**, 112 (10), 2051, https://doi.org/10.1002/bit.25624.

[12] R.-B. Song, Y. Wu, Z.-Q. Lin, J. Xie, C. H. Tan, J. S. C. Loo, B. Cao, J.-R. Zhang, J.-J. Zhu, Q. Zhang. *Angew. Chem. Int. Ed.* **2017**, 56, 10516-10520.

[13] Y.-Y. Yu, Y.-Z. Wang, Z. Fang, Y.-T. Shi, Q.-W. Cheng, Y.-X. Chen, W. Shi, Y.-C. Yong, *Nat. Commun.* **2020**, 11, 4087.

[14] H. R. Luckarift, S. R. Sizemore, J. Roy, C. Lau, G. Gupta, P. Atanassov, G. R. Johnson. *Chem. Commun.* **2010,** 46, 6048-6050.

[15] Y.-C. Yong, Y.-Y. Yu, X. Zhang, H. Song, *Chem. Int. Ed.* **2014,** 53, 4480-4483.

[16] S. Zhao, Y. Li, H. Yin, Z. Liu, E. Luan, F. Zhao, Z. Tang, S. Liu. *Sci. Adv.* **2015**, 1(10), e1500372.

[17] H.-F. Cui, L. Du, P.-B. Guo, B. Zhu, J. H. T. Luong, *J. Power Sources* **2015***,* 283, 46-53.

[18] Y. Qiao, S. J. Bao, C. M. Li, X. Q. Cui, Z. S. Lu, J. Guo, *ACS Nano* **2008**, 2, 113-119.

[19] L. Zou, Y. Qiao, C. Y. Zhong, C. M. Li, *Electrochim. Acta* **2017,** 229, 31-38.

[20] Y. Hou, H. Y. Yuan, Z. H. Wen, S. M. Cui, X. R. Guo, Z. He, J. H. Chen. *J. Power Sources* **2016,** 307, 561-568.

[21] Z. Lv, Y. Chen, H. Wei, F. Li, Y. Hu, C. Wei, C. Feng. *Electrochim. Acta* **2013,** 111, 366-373.

[22] X. S. Wu, Z. Z. Shi, L. Zou, C. M. Li, Y. Qiao. *J. Power Sources* **2018***,* 378, 119-124.

[23] Liu, Y.-C.; Hung, Y.-H.; Liu, S.-F.; Guo, C.-H.; Liu, T.-Y.; Sun, C.-L.; Chen, H.-Y. *Sust. Energy Fuels* **2020,** 4 (10), 5339-5351
